# Role of Cell Wall Polyphosphates in Phosphorus Transfer at the Arbuscular Interface in Mycorrhizas

**DOI:** 10.3389/fpls.2021.725939

**Published:** 2021-09-20

**Authors:** Cuc Thi Nguyen, Katsuharu Saito

**Affiliations:** ^1^Department of Bioscience and Food Production Science, Interdisciplinary Graduate School of Science and Technology, Shinshu University, Nagano, Japan; ^2^Faculty of Agriculture and Forestry, Dalat University, Dalat, Vietnam

**Keywords:** arbuscular mycorrhizal fungi, arbuscule, acid phosphatase, cell wall, H^+^-ATPase, periarbuscular space, polyphosphate, two-compartment system

## Abstract

Arbuscular mycorrhizal fungi provide plants with soil mineral nutrients, particularly phosphorus. In this symbiotic association, the arbuscular interface is the main site for nutrient exchange. To understand phosphorus transfer at the interface, we analyzed the subcellular localization of polyphosphate (polyP) in mature arbuscules of *Rhizophagus irregularis* colonizing roots of *Lotus japonicus* wild-type (WT) and H^+^-ATPase *ha1-1* mutant, which is defective in phosphorus acquisition through the mycorrhizal pathway. In both, the WT and the *ha1-1* mutant, polyP accumulated in the cell walls of trunk hyphae and inside fine branch modules close to the trunk hyphae. However, many fine branches lacked polyP. In the mutant, most fine branch modules showed polyP signals compared to the WT. Notably, polyP was also observed in the cell walls of some fine branches formed in the *ha1-1* mutant, indicating phosphorus release from fungal cells to the apoplastic regions. Intense acid phosphatase (ACP) activity was detected in the periarbuscular spaces around the fine branches. Furthermore, double staining of ACP activity and polyP revealed that these had contrasting distribution patterns in arbuscules. These observations suggest that polyP in fungal cell walls and apoplastic phosphatases may play an important role in phosphorus transfer at the symbiotic interface in arbuscules.

## Introduction

Phosphorus is a crucial element for plant growth and development. Terrestrial plants absorb P as orthophosphate (Pi) from the soil solution. However, soil Pi is mainly present in immobile forms that are not directly available to plants (Pierzynski et al., 2005). Plants have evolved several mechanisms to overcome Pi-deficient conditions. One of the oldest adaptive strategies include formation of mutualistic symbiosis with arbuscular mycorrhizal (AM) fungi (Raghothama, 1999; Delaux et al., 2015; Field and Pressel, 2018). During symbiosis, host plants can acquire soil Pi *via* two pathways, the mycorrhizal pathway and the direct pathway. The mycorrhizal pathway is a route *via* AM fungal hyphae (Smith et al., 2003, 2011). In the direct pathway, Pi is directly taken up by plant roots. The mycorrhizal pathway is usually activated even in non-responsive AM plants, for which AM fungal colonization does not positively affect growth or P nutrition (Smith et al., 2003, 2004). The overall flow of P through the mycorrhizal pathway includes: (1) P uptake from soil by AM fungal extraradical hyphae that extend far beyond the P depletion zone surrounding the root system; (2) long-distance P translocation through extraradical and intraradical hyphae; (3) P release from arbuscules that are the highly branched fungal structure formed in root cortical cell; and (4) P transfer into the host plant cells (Saito and Ezawa, 2016; Ezawa and Saito, 2018).

AM fungi absorb Pi from soil through the Pi transporters localized on the plasma membrane of extraradical hyphae (Harrison and van Buuren, 1995; Maldonado-Mendoza et al., 2001; Benedetto et al., 2005; Fiorilli et al., 2013; Xie et al., 2016). The Pi taken up is rapidly converted into polyphosphate (polyP) that is sequestered into tubular vacuoles (Rasmussen et al., 2000; Uetake et al., 2002; Ezawa et al., 2004; Viereck et al., 2004; Kuga et al., 2008; Hijikata et al., 2010; Kikuchi et al., 2014, 2016; Nayuki et al., 2014). PolyP is a linear polymer of Pi linked by high-energy phosphoanhydride bonds and likely to be synthesized by the vacuolar transporter chaperone (VTC) complex consisted of subunits VTC1, VTC2, and VTC4 using ATP as substrate (Tani et al., 2009; Tisserant et al., 2012; Kikuchi et al., 2014; Ezawa and Saito, 2018). P translocation in extraradical hyphae is bidirectional. However, the net flow of P toward intraradical hyphae that are the main sink for P (Nielsen et al., 2002; Viereck et al., 2004; Hijikata et al., 2010). A water potential gradient in AM fungal hyphae has been proposed as a driving force for long-distance translocation of P (Kikuchi et al., 2016). Once polyP is delivered to the intraradical hyphae, its chain length is shortened (Solaiman et al., 1999; Viereck et al., 2004; Ohtomo and Saito, 2005; Takanishi et al., 2009) in a reaction possibly catalyzed by fungal endopolyphosphatases. This depolymerized polyP in arbuscules may be a significant source of P for host plants (Solaiman and Saito, 2001; Takanishi et al., 2009). There is no doubt that arbuscules play a vital role in P supply through P release from fungal cells. However, the mechanism of P export from arbuscules remains unclear.

Conversely, the molecular mechanisms underlying plant’s uptake of Pi released from arbuscules are well documented. Plant Pi transporter genes that are upregulated during AM symbiosis have been identified in many plant species reviewed in Chiu and Paszkowski (2019). In particular, the AM-specific transporter genes of the *Medicago truncatula PT4*/*Oryza sativa PT11* clade are conserved in all AM vascular plants (Yang et al., 2012; Delaux et al., 2015) and are strongly expressed in arbuscule-containing cortical cells (Harrison et al., 2002; Glassop et al., 2005; Nagy et al., 2005; Yang et al., 2012). Arbuscules are surrounded by host-derived periarbuscular membrane (PAM; Smith and Smith, 1990, 2011; Harrison, 1999) with localized MtPT4/OsPT11 proteins (Harrison et al., 2002; Kobae and Hata, 2010; Pumplin et al., 2012). Mutation of *MtPT4*, *OsPT11*, and their orthologs severely impairs symbiotic Pi uptake, indicating that AM-specific Pi transporters play a central role in the mycorrhizal pathway (Javot et al., 2007; Yang et al., 2012; Willmann et al., 2013; Xie et al., 2013; Watts-Williams et al., 2015). These Pi transporters are classified as H^+^/Pi symporters and require H^+^ electrochemical potential gradient across the PAM for Pi uptake from an apoplastic interface between the plant and fungus, the periarbuscular space (PAS), into a plant cell. PAS is an acidified compartment (Guttenberger, 2000) in which H^+^-ATPase activity has been observed (Marx et al., 1982; Gianinazzi-Pearson et al., 1991). The *Medicago* HA1, rice HA1, and tomato HA8 proteins have been identified as AM-specific H^+^-ATPases that are responsible for generating the H^+^ gradient across PAS, contributing to symbiotic Pi uptake by AM-specific Pi transporters (Krajinski et al., 2014; Wang et al., 2014; Liu et al., 2020).

P release from AM fungus to PAS in arbuscule-containing cortical cells is a critical step in the mycorrhizal pathway. Mathematical modeling of AM fungus-plant nutrient exchange dynamics predicts that P is exported as Pi by fungal H^+^-coupled Pi transporters localized in the plasma membrane of arbuscules (Schott et al., 2016; Dreyer et al., 2018). H^+^-coupled Pi transporters expressed in intraradical hyphae have indeed been identified in AM fungi (Balestrini et al., 2007; Fiorilli et al., 2013; Xie et al., 2016). However, no evidence was found for Pi efflux into the PAS *via* these transporters. Ezawa and Saito (2018) postulated the involvement of the AM fungal SYG proteins (named after suppressor of yeast gpa1) in P release from arbuscules based on evidence that animal and plant SYG homologs mediate Pi export (Arpat et al., 2012; Giovannini et al., 2013). An alternative hypothesis is that polyP is directly exported to interfacial apoplasts around arbuscules (Saito and Ezawa, 2016). This hypothesis is supported by the observation that polyP is distributed in the cell walls of germ tubes and extraradical hyphae of AM fungi (Werner et al., 2007; Kuga et al., 2008). However, the precise distribution of polyP in arbuscules remains unclear. In this study, we determined the subcellular localization of polyP in arbuscules using 4′,6-diamidino-2-phenylindole (DAPI) staining (Tijssen et al., 1982) and by enzyme-affinity labeling with the polyP binding domain (PPBD) of *Escherichia coli* exopolyphosphatase PPX1 (Saito et al., 2005) to elucidate the role of polyP in P transfer at the symbiotic interface. In this analysis, we used *Lotus japonicus* with an AM-specific H^+^-ATPase *HA1* mutation to analyze P dynamics in mature arbuscules. *Medicago*, rice, and tomato *ha1* mutants, similar to the *pt4*/*pt11* mutants, show a decrease in mature arbuscules due to degeneration of premature arbuscules (Krajinski et al., 2014; Wang et al., 2014; Liu et al., 2020). However, there is considerable variation in phenotype depending on the plant species and mutant alleles. Here, we analyzed polyP distribution in the *L. japonicus ha1-1* mutant that shows a relatively mild defect in arbuscule formation.

## Materials and Methods

### Biological Materials and Growth Conditions

**Figure 1:**
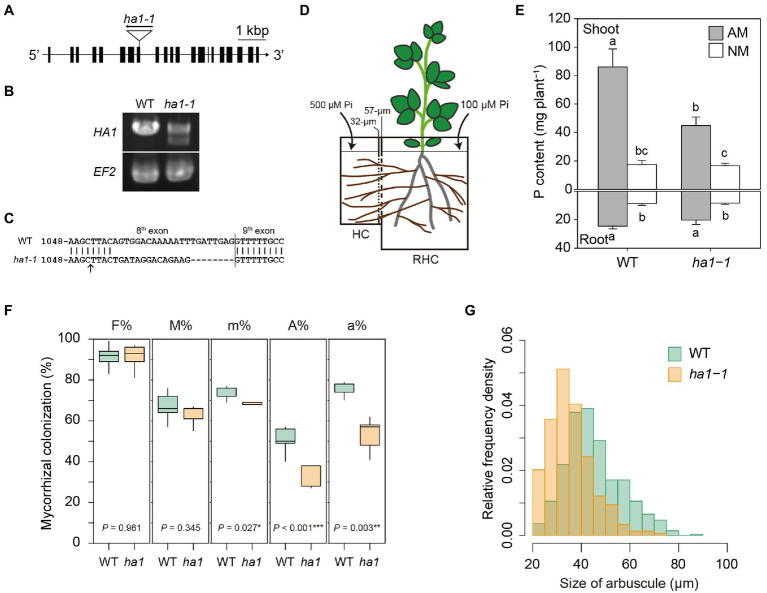
Phenotype of the *ha1-1* mutant cultivated in the two-compartment system. **(A)** Exon-intron structure of the *HA1* gene, including the *ha1-1 LORE1* insertion site. **(B)** RT-PCR analysis of *HA1* in wild-type (WT) and *ha1-1* roots colonized by *Rhizophagus irregularis*. The elongation factor 2 (*EF2*) gene was amplified as the loading control. PCR was performed using 10ng of cDNA as a template with 35cycles. Similar results were obtained in three independent experiments. **(C)** Sequence comparison between WT and the *ha1-1 HA1* cDNAs showing frameshift indels in the *ha1-1* mutant transcript. The arrow indicates the *LORE1* insertion site. **(D)** The two-compartment system: the root-hyphal (RHC) and hyphal (HC) compartments were separated by 32- and 57-μM nylon meshes, allowing only hyphae of arbuscular mycorrhizal fungi to pass into the HC. The compartments were filled with autoclaved river sand. RHC was supplied with a half-strength Hoagland’s solution containing a low phosphate (Pi) concentration (100μM). HC was supplied with a nutrient solution with a high (500μM) concentration of phosphate. **(E)** P content in shoots and roots of arbuscular mycorrhizal (AM) and non-mycorrhizal (NM) plants 4weeks after inoculation. Error bars show SE (*n*=5). For each plant part, bars topped by the same letter do not differ significantly at *p*<0.05 on Tukey’s multiple comparisons test. **(F)** Arbuscular mycorrhizal fungal colonization in WT and *ha1-1* roots. Mycorrhizal colonization was assessed according to method suggested by Trouvelot et al. (1986) after trypan blue staining. F%, frequency of mycorrhiza in the root system; M%, intensity of mycorrhizal colonization in the root system; m%, intensity of mycorrhizal colonization in the root fragments; A%, arbuscule abundance in the root system; and a%, arbuscule abundance in mycorrhizal parts of root fragments. The boxes show the first quartile, the median, and the third quartile; the whiskers reach 1.5 times the interquartile range (*n*=5). *p*-values are based on Student’s *t*-tests (^*^, *p*<0.05; ^*^, *p*<0.01; ^**^, *p*<0.001). **(G)** Relative frequency density of arbuscule size. The lengths of the major axis of 549 WT and 297*ha1-1* arbuscules randomly selected from >20μM-long arbuscules stained with wheat germ agglutinin conjugated with Oregon Green 488 were measured. According to the two-sample Kolmogorov–Smirnov test, the size distribution was significantly different between the plant genotypes (*p*<0.001).

The *L. japonicus* homozygous *ha1-1* mutant line with a *LORE1* insertion in the *HA1* gene (gene ID: LotjaGi3g1v0066100.1 in the Lotus Base Gifu v1.2) and the wild-type (WT) segregant were selected from a heterozygous *LORE1* insertion line (plant ID: 30006854) that was obtained from Lotus Base.^1^ Genotyping was performed using *HA1*-specific primers (Supplementary Table S1) combined with the P2 internal *LORE1* primer (Fukai et al., 2012; Urbański et al., 2012). A two-compartment culture system consisting of root-hyphal (RHC) and hyphal (HC) compartments was used to cultivate plants (Figure 1). The two compartments were separated by a three-layered barrier (Kobae et al., 2014) comprising an RHC filter (57μM opening), a medial mesh (1mM in thickness; 2mM opening), and an HC filter (32μM opening), which prevented plant roots from passing through but allowed AM fungal hyphae to pass. The compartments were filled with autoclaved river sand (particle size, 0.5–2.0mM). *L. japonicus* seeds were surface sterilized with a hypochlorite solution, sown on a wetted filter paper in a Petri dish, and germinated at 26°C for 2days in dark and 3days in light. Seedlings were transplanted to RHC and inoculated with 500 spores of *Rhizophagus irregularis* DAOM 197198 (Mycorise, Premier Tech, Rivière-du-Loup, Canada) per plant. The inoculated and non-inoculated plants were grown in a growth chamber (photoperiod: 16h, temperature: 26°C, and photosynthetic photon flux density: 150μMolm^−2^ s^−1^) for 4weeks. RHC was supplied with a half-strength Hoagland’s solution containing a low concentration of KH_2_PO_4_ (100μM) every 2days to promote AM colonization. HC was provided with a high-nutrient solution with 500μM KH_2_PO_4_ to enhance P translocation from the HC *via* AM fungal hyphae. The amount of applied fertilizer that did not flow from the HC into the RHC was determined before cultivation.

### Cryostat Sectioning

Fresh mycorrhizal roots from at least three independent plants were cut into 5–10mM fragments, placed in plastic molds, and covered with the O.C.T. compound (Sakura Finetek, Tokyo, Japan). The roots were frozen in dry ice-isopentane and stored at −80°C. The frozen roots were sectioned longitudinally at a thickness of 25–30μM using a cryostat (Leica, Wetzlar, Germany). Cryosections were placed on an adhesive glass slide (Frontier, Matsunami, Osaka, Japan) and dried at room temperature for 60min. These sections were used for DAPI staining and enzyme cytochemistry of phosphatase activity.

### High-Pressure Freezing and Freeze-Substitution

Mycorrhizal roots from at least three independent plants of each genotype were cut into 5mM fragments using a razor blade in a Petri dish on ice. The root fragments were placed in aluminum specimen carriers with 0.05M sucrose as a cryoprotectant and then frozen using a Leica EM PACT2 high-pressure freezer. The frozen samples were temporarily maintained in liquid nitrogen and transferred into vials containing 1% glutaraldehyde in anhydrous acetone that was precooled to −80°C. The samples were freeze-substituted at −80°C for 48h and gradually warmed to 4°C over a period of 21h using a Leica EM AFS2. Roots were washed twice with anhydrous acetone for 15min and twice with propylene oxide for 5min at room temperature. The samples were infiltrated with Spurr’s resin: propylene oxide mixture of ratios 1:2, 1:1, 2:1, 3:1, and 4:1 for 1h in each mixture sequentially (Spurr’s resin from Polysciences, PA, United States). Final soak was thrice in pure resin for 3h, and samples were polymerized at 70°C for 12h. The embedded roots were trimmed and sectioned longitudinally using an ultramicrotome. For fluorescence microscopy, semithin sections, approximately 200nm thick, were cut with a knife (XAC, SYNTEC, Yokohama, Japan) and placed on an adhesive glass slide. For transmission electron microscopy (TEM), ultrathin sections of approximately 90nm thickness were cut with a diamond knife (DiATOME, PA, United States) and picked up on nickel grids. These sections were immediately processed for polyP labeling.

### PolyP Detection by DAPI Staining

Cryosections of fresh roots and semithin sections of resin-embedded roots were used for DAPI staining. Cryosections were washed gently with 70% ethanol, 25% ethanol, and distilled water (DW) sequentially for 3min in each solution. Semithin sections were etched with 0.2% sodium ethoxide in ethanol for 5min and then washed twice with 100% ethanol, 70% ethanol, and 25% ethanol sequentially for 3min in each solution. Root samples were stained with 80μgml^−1^ DAPI in 70% ethanol for 60min at room temperature in the dark. After washing with 70% ethanol and DW, the sections were mounted in DW and observed within 1h. Fluorescence microscopy was performed using an Axio Imager D1 microscope (Carl Zeiss, Jena, Germany). DAPI-polyP fluorescence was excited with UV light, and the emitted fluorescence was detected using a long-pass filter, LP420. Digital images were captured with a digital CCD camera (AxioCam MRc5, Carl Zeiss) operated with AxioVision (Carl Zeiss). After randomly selecting images of mature arbuscules, the areas showing the DAPI-polyP signal in the arbuscules were measured using ImageJ software (Schneider et al., 2012).

### PolyP Detection by Enzyme Affinity Labeling With PPBD

PolyP labeling using *E. coli* PPBD was performed according to the method described by Saito et al. (2005) and Kuga et al. (2008) with some modifications. For fluorescence microscopy, semithin sections of the resin-embedded roots were etched with 0.2% sodium ethoxide in ethanol for 5min and then washed with 0.05% sodium ethoxide followed by 100% ethanol, 50% ethanol, and DW wash. The sections were blocked with 1% bovine serum albumin (BSA) in Tris-buffered saline (TBS) containing low concentrations of salts (TBS-low salt; 25mM Tris-HCl pH 7.4, 13.7mM sodium chloride, and 0.27mM potassium chloride) for 10min. Sections were first incubated in a mixture of 20μgml^−1^ PPBD, 10μgml^−1^ mouse anti-Xpress epitope antibody (#R910-25, Thermo Fisher Scientific, MA, United States), TBS-low salt, and 1% BSA for 2h at room temperature. The sections were then washed with TBS-low salt buffer containing 0.05% Triton X-100, followed by TBS-low salt. Samples were incubated with a goat anti-mouse IgG antibody conjugated with Alexa Fluor 488 (#A28175, Thermo Fisher Scientific) diluted to ratio 1:100 in TBS-low salt containing 1% BSA for 1h at room temperature. The sections were washed with TBS-low salt containing 0.05% Triton X-100, followed by TBS-low salt and DW. Alexa Fluor 488 fluorescence was excited with a band-pass filter BP470/40, and the emitted fluorescence was detected with BP525/50 using an epifluorescence microscope Axio Imager D1. Digital images were captured with a digital CCD camera (AxioCam MRm, Carl Zeiss) operated with AxioVision.

For TEM observation, ultrathin sections of the resin-embedded roots were etched with 0.2% sodium ethoxide in ethanol for 5min. They were then washed with 0.02% sodium ethoxide, followed by 100% ethanol, 50% ethanol, and DW wash. Specimens were blocked with 1% BSA in phosphate-buffered saline (PBS) containing low concentrations of salts (PBS-low salt; 10mM phosphate buffer pH 8.4, 13.7mM sodium chloride, and 0.27mM potassium chloride). They were then incubated in a mixture of 20μgml^−1^ PPBD, 10μgml^−1^ mouse anti-Xpress epitope antibody, PBS-low salt, and 1% BSA, and washed with PBS-low salt buffer containing 0.05% Triton X-100, followed by PBS-low salt. Samples were incubated with a goat anti-mouse IgG antibody conjugated with 6nm colloidal gold (#115-195-146, Jackson ImmunoResearch Laboratories, PA, United States) diluted 1:20 in PBS-low salt buffer containing 1% BSA for 1h at room temperature. The samples were washed with PBS-low salt containing 0.05% Triton X-100, followed by PBS-low salt and DW wash. Specimens were stained with 50% TI-Blue (Nisshin EM, Tokyo, Japan) for 10min, followed by lead citrate staining for 5min, and observed using a TEM (JEOL, Tokyo, Japan) at an accelerating voltage of 80kV. Negative controls were prepared by incubating sections without PPBD. Another negative control was prepared by incubating sections with an excessive competitor (100mM tripolyphosphate) during the first reaction.

### Enzyme Cytochemistry of Phosphatase Activity

Acid phosphatase (ACP) and neutral phosphatase (NTP) activity was detected using TEM with a cerium-based method (Dreyer et al., 2008) with some modifications. Root fragments (5–10mM) were pre-fixed with 2.5% glutaraldehyde in 100mM cacodylate buffer (pH 7.0) for 2h on ice. The fragments were washed twice with cacodylate buffer for 30min. Roots were further cut into 0.5mM fragments. The fragments were sequentially incubated in a pre-incubation buffer [2mM CeCl_3_ in 100mM acetate buffer (pH 4.6) and 100mM Tris-HCl buffer (pH 7.4) for acid and NTP activity, respectively] for 1h, a reaction buffer (1mM β-glycerophosphate and 2mM CeCl_3_ in acetate or Tris-HCl buffer) for 30min, a pre-incubation buffer for 15min, and an acetate or Tris-HCl buffer for 15min at 37°C. Samples were post-fixed with 1% OsO_4_ in cacodylate buffer for 2h on ice, followed by washing with DW thrice for 5min. Roots were dehydrated with an ethanol series (20, 50, 70, 90, and 95% once and 100% thrice) for 20min each at 4°C and incubated twice with propylene oxide for 5min at room temperature. The samples were infiltrated with Spurr’s resin:propylene oxide (1:2, 1:1, 2:1, 3:1, and 4:1) for 1h in each step and thrice with pure resin for 3h, and polymerized at 70°C for 12h. Ultrathin sections on copper grids were stained with gadolinium acetate (Nakakoshi et al., 2011) and lead citrate and observed using a TEM at an accelerating voltage of 80kV. Controls were prepared by incubating sections without β-glycerophosphate in the reaction buffer and without 2mM CeCl_3_ in the pre-incubation and reaction buffers.

The localization of phosphatase activity was also analyzed by fluorescence microscopy. Cryosections of mycorrhizal roots were fixed with 0.25% glutaraldehyde in PBS (pH 7.4) for 8min at 4°C. They were then permeabilized by immersion in 25% ethanol in 100mM acetate buffer (pH 4.6) and 100mM Tris-HCl buffer (pH 7.4) for several seconds for ACP and NTP activity, respectively, and washed twice with the same buffer. The sections were incubated with 25μM ELF97 phosphatase substrate (Thermo Fisher Scientific) in either acetate or Tris-HCl buffer for 30min at room temperature in the dark and washed with the same buffer without the substrate. Fluorescence of the ELF97 reaction product was excited with UV, and the emitted fluorescence was detected with a long-pass filter, LP420, using an epifluorescence microscope Axio Imager D1 microscope.

For dual labeling of polyP accumulation and phosphatase activity, cryosections of mycorrhizal roots were sequentially washed with 70% ethanol, 25% ethanol, and DW. First, sections were labeled with 80μgml^−1^ DAPI in either 100mM acetate buffer (pH 4.6) or 100mM Tris-HCl buffer (pH 7.4) for 60min at room temperature in the dark. The samples were then gently washed in either acetate buffer or Tris-HCl buffer. Subsequently, sections were incubated with 25μM ELF97 in acetate buffer or Tris-HCl buffer for 30min at room temperature in the dark. After the second labeling, the sections were mounted in DW, covered with a glass coverslip, and observed within 30min. The fluorescence of the DAPI-polyP complex and the ELF97 reaction product was excited with UV, and the emitted fluorescence was detected with a long-pass filter, LP420, using the Axio Imager D1 microscope. Digital images were captured using an AxioCam MRc5 operated with AxioVision.

### AM Fungal Colonization

Roots were cleared in a 10% (w/v) KOH solution at 90°C for 10min, acidified with a 2% (v/v) HCl solution for 5min, and then stained with 0.05% trypan blue in lactic acid at 90°C for 10min. AM colonization was assessed using the method described by Trouvelot et al. (1986) with some modifications. Approximately 10 root fragments per plant were mounted on a slide and observed under a light microscope. The intensity of AM fungal colonization and arbuscule abundance in a field of view (diameter: 1.28mM) was categorized into six and three classes, respectively. AM colonization parameters (F%, M%, m%, A%, and a%) were calculated based on the scores in 100 fields of view. To visualize the fine structure of AM fungal colonization, roots were also stained with wheat germ agglutinin (WGA) conjugated with Oregon Green 488 (Kojima et al., 2014). Fluorescent images of the mycorrhizal roots were captured using an Axio Imager D1 microscope equipped with a digital CCD camera AxioCam MRc5. The length of the major axis of arbuscules was measured with the AxioVision software.

### Plant P Analysis

The plants were divided into shoots and roots and dried at 70°C for 48h and were weighed after cooling in a desiccator. Dried samples were digested with sulfuric acid and hydrogen peroxide at 200°C for 2h. P concentrations in the samples were determined using the acid-molybdate blue method (Watanabe and Olsen, 1965). The P content was calculated by multiplying each sample’s dry weight by their P concentrations.

### Gene Expression Analysis

Total RNA was isolated from roots using RNAiso Plus (Takara Bio, Shiga, Japan) combined with Fruit-mate (Takara Bio) according to the manufacturer’s instructions. Contaminating genomic DNA was eliminated using a TURBO DNA-*free* kit (Thermo Fisher Scientific). cDNA was synthesized using a ReverTra Ace qPCR RT Kit (Toyobo, Osaka, Japan). Quantitative PCR was conducted using a StepOne Real-Time PCR System (Thermo Fisher Scientific) with a THUNDERBIRD SYBR qPCR Mix (TOYOBO). The primers used for qRT-PCR are listed in Supplementary Table S1. The *L. japonicus* elongation factor 2 gene and *R. irregularis EF1β* (Kobae et al., 2015) genes were used as internal controls for the expression analysis of plant and AM fungal genes, respectively. Melting curve analysis confirmed the presence of single peaks. Relative expression levels were calculated using the 2^−ΔCt^ method (Schmittgen and Livak, 2008). All the reactions were performed using three biological replicates. RT-PCR was performed using *HA1*-specific primers (Supplementary Table S1) and 10ng of cDNA template to confirm homozygous *LORE1* insertion lines at the transcript level, and the amplified products were sequenced.

### Statistical Analyses

All statistical analyses were performed using the software R (version 4.0.0). The mycorrhizal colonization rate and relative gene expression values were logit-transformed (ln[p/1−p]) and log-transformed, respectively, to avoid violating normality and homoscedasticity assumptions. To test the differences in mycorrhizal colonization and gene expression between WT and *ha1-1* mutants, data were analyzed using the Student’s *t*-test. Tukey’s HSD test was performed for multiple comparisons of P contents of plants. Relative frequency densities of arbuscule size and the percentage of arbuscule area showing DAPI-polyP signal were calculated using the MASS package in the software. The size distributions of WT and *ha1-1* mutants were compared using a two-sample Kolmogorov–Smirnov test. The proportion of mature and collapsed arbuscules in WT and *ha1-1* was analyzed using Fisher’s exact test.

## Results

### AM Phenotype of the *ha1-1* Mutant in *Lotus japonicus*

First, we investigated the effect of the mutation of *HA1* on P acquisition through the mycorrhizal pathway. A *L. japonicus* homozygous line carrying a *LORE1* insertion in exon 8 of the *HA1* gene was selected to obtain a *ha1* mutant, *ha1-1* (Figure 1A). RT-PCR analysis revealed that although *HA1* transcripts were observed in AM roots of the *ha1-1* mutant (Figure 1B), they had indels caused by putative abnormal splicing that created a frameshift and a premature stop codon 13 amino acids after the insertion (Figure 1C). To determine whether the *HA1* mutation affects P acquisition *via* the mycorrhizal pathway, we examined the P nutrition of the *ha1-1* mutant using a two-compartment system consisting of RHC and HC (Figure 1D). Under uninoculated conditions, plant’s P nutrition did not differ between WT and *ha1-1* plants (Figure 1E). In the presence of AM fungi, P content increased in both genotypes. However, the positive effects on shoot P content were lower in the *ha1-1* mutant (average increase of 268%) than in the WT (average increase of 492%). These data demonstrate that P transfer from the AM fungus to the host *via* the mycorrhizal pathway was partially impaired in the *ha1-1* mutant. To examine the effects of *ha1-1* on hyphal development within roots, we assessed AM fungal colonization by staining mycorrhizal roots with trypan blue and WGA–Oregon Green 488. Quantitative analysis revealed that arbuscule densities (A% and a%) were moderately reduced in the mutant relative to the WT 4weeks post-inoculation (Figure 1F). Furthermore, the arbuscules formed in *ha1-1* mutant roots were slightly smaller than those in WT roots (Figure 1G). However, the range of arbuscule sizes almost overlapped between the two genotypes. These results suggest that the *HA1* mutation induced a phase transition of the arbuscule life cycle from mature to degrading stage.

### PolyP Localization in Arbuscules – DAPI Staining

**Figure 2:**
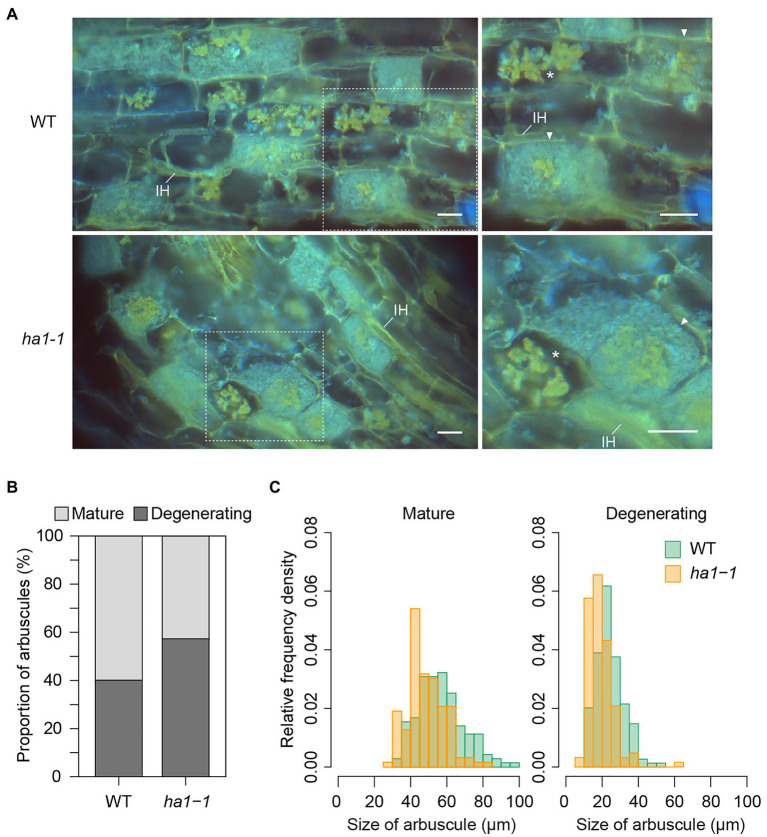
Mature and degenerating arbuscules stained with 4′,6-diamidino-2-phenylindole (DAPI). **(A)** DAPI staining of arbuscules in wild-type (WT) and *ha1-1* roots of *Lotus japonicus*. Mycorrhizal roots were harvested 4weeks after inoculation with *Rhizophagus irregularis*. Cryosections of fresh roots were stained with DAPI and observed using an epifluorescence microscope. The DAPI-polyP complex emits yellow fluorescence under UV irradiation. Blue fluorescence suggests non-specific DAPI binding to fungal hyphae. Right panels show the magnified images of dotted areas in the left panels. Mature arbuscules (arrowheads) and degenerating arbuscules (asterisks) are visible. IH: intraradical hypha. Scale bar=20μM. **(B)** Proportion of mature and degenerating arbuscules in WT and the *ha1-1* mutant. Small, shrunk arbuscules that entirely show intense yellow fluorescence by DAPI staining were considered as degenerating arbuscules. Mature arbuscules fully occupy plant cortical cells. The proportion was significantly different based on Fisher’s exact test (*p*<0.001, *n*=499 and 562 in WT and *ha1-1*, respectively). **(C)** Relative frequency density of the size of mature and degenerating arbuscules formed in WT and *ha1-1*. The lengths of the major axis of arbuscules stained with DAPI were measured. The size distribution was significantly different between the plant genotypes according to the two-sample Kolmogorov-Smirnov test in each arbuscule type (*p*<0.001). Mature arbuscule: *n*=143 and 146; degenerating arbuscule: *n*=149 and 125 in WT and *ha1-1*, respectively.

We analyzed polyP accumulation in arbuscules by DAPI staining. Once DAPI binds to polyP, the complex emits yellow fluorescence under UV irradiation (Tijssen et al., 1982). Mycorrhizal root cryosections were DAPI-stained after polyP fixation and cell permeabilization with ethanol. We observed arrays of fully-developed arbuscules (mature arbuscules) and the patchy colonization of degenerating arbuscules which are small arbuscules with shrunken fine branches (Figure 2A). In most mature arbuscules formed in cortical cells, the fluorescent DAPI-polyP complex signal was confined to the central region, irrespective of the plant’s genotype. The other region within the arbuscules, containing fine branches, exhibited non-specific blue fluorescence. In contrast, the entire hyphae of the degenerating arbuscules were stained with DAPI and fluoresced yellow. This type of arbuscules accounted for 57% of the total arbuscules formed in *ha1-1* mutants, which was 1.4-fold higher than that in the WT (Figure 2B). This result prompted us to examine whether the shift in size distribution toward small arbuscules in the mutant, as observed by WGA staining was due to the increased number of degenerating arbuscules. We measured the size of mature and degenerating arbuscules distinguished by the DAPI staining pattern. Overall, WGA staining reduced arbuscule size relative to DAPI, possibly due to cell shrinkage caused by fixation with Farmer’s solution which contained ethanol and acetic acid (Figure 1G and Figure 2C). In both arbuscule types, the size distribution almost overlapped between the WT and the *ha1-1* mutant, but the median length was 5–8μM shorter in the mutant (Figure 2C). Our DAPI staining data also suggested that *HA1* mutations caused early arbuscule degeneration.

**Figure 3:**
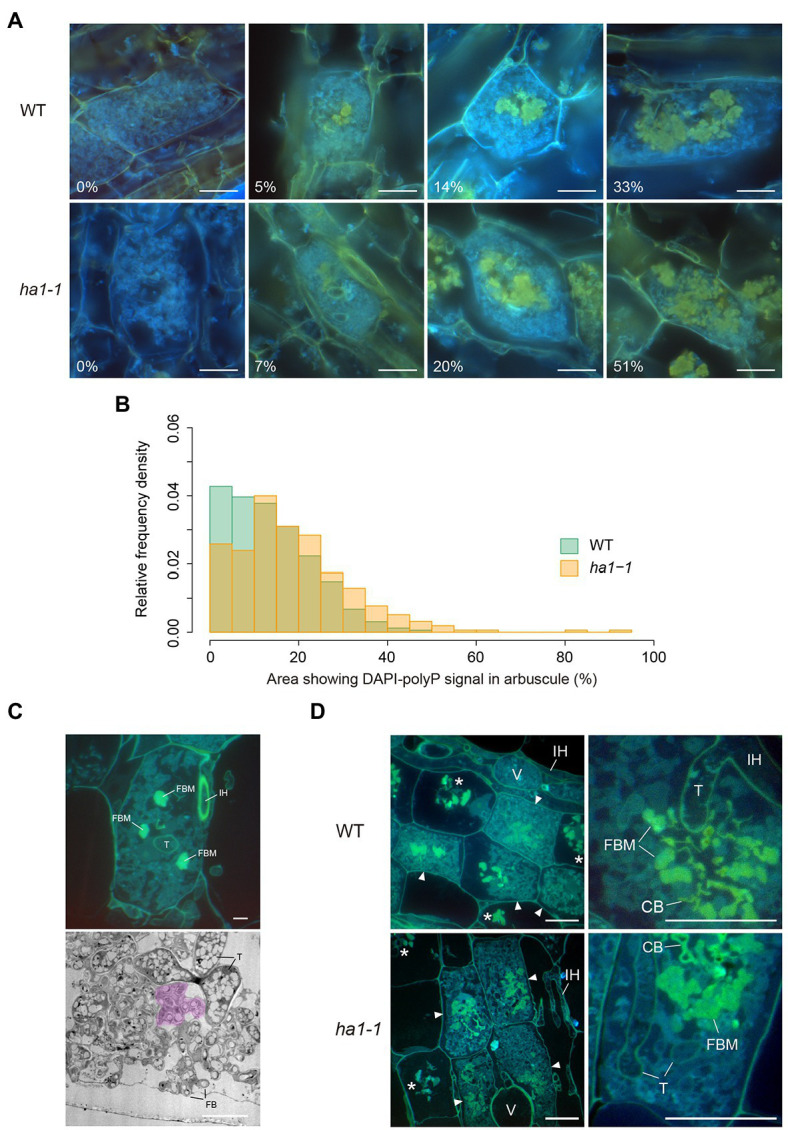
Localization of polyphosphate (polyP) by DAPI staining. **(A)** Representative types of mature arbuscules stained with DAPI. Mycorrhizal roots of *L. japonicus* were harvested 4weeks after inoculation with *R. irregularis*. Yellow fluorescence is observed in the central part of arbuscules. Percentages of arbuscule area showing yellow fluorescence signals are shown. Scale bars=20μM. **(B)** Relative frequency density of the area showing DAPI-polyP signal. The percentage of arbuscule area with signal was calculated. In total, 323 and 310 mature arbuscules were measured for wild-type (WT) and *ha1-1*, respectively. According to the two-sample Kolmogorov–Smirnov test, the size distribution was significantly different between the plant genotypes (*p*<0.001). **(C)** Fine branch modules (FBM) in arbuscules. In the upper panel, semithin sections of mature arbuscules in resin-embedded WT roots were stained with DAPI and observed using an epifluorescence microscope. Strong yellow fluorescence was observed in several fine branch modules, a set of connected fine branches. The lower panel shows a transmission electron micrograph of fine branches and trunk hyphae of an arbuscule colonized in a WT cortical cell. Acid phosphatase (ACP) activity was detected by electron microscopic enzyme cytochemistry as black precipitates of the reaction product (cerium phosphate). The region colored in magenta is a putative FBM in which fine branches form a cluster. FB: fine branch, FBM: fine branch module, IH: intraradical hypha, T: trunk hypha. Scale bars=5μM. **(D)** DAPI staining of semithin sections of resin-embedded mycorrhizal roots. In the left panels, mature arbuscules (arrowheads) and degenerating arbuscules (asterisks) are shown. Right panels show magnified images of mature arbuscules. Fine branch modules (clusters of connected branches), trunk hyphae cell walls, and collapsed branches display yellow fluorescence. CB: collapsed branch, FBM: fine branch module, IH: intraradical hypha, T: trunk hypha, and V: vesicle. Scale bars=20μM.

Next, we focused on the distribution of polyP in mature arbuscules. It should be noted that the size of the DAPI-stained region in the arbuscules varied (Figure 3A). Quantitative analysis revealed that the distribution of the yellow fluorescent area relative to the entire mature arbuscule significantly differed between the WT and *ha1-1* (Figure 3B). In the WT, the most abundant size class was 0–5%; arbuscules with large DAPI-stained regions were infrequent. In contrast, the size distribution in the *ha1-1* mutant peaked at 10–15% with a higher right tail than the WT, indicating an expansion of the DAPI-polyP positive region.

To obtain more precise polyP localization in mature arbuscules, we analyzed semithin sections of resin-embedded AM roots. Arbuscules develop with repeated branching and narrowing of hyphal diameter from a thick trunk hypha to fine branches. Here, we define the fine branch module as a set of connected branches (Figure 3C). For arbuscules generated in WT roots, yellow fluorescence was detected in some fine branch modules located in the vicinity of the trunk hyphae (Figure 3D). The fluorescent signal was also visible around the periphery of the trunk hyphae where the cell wall was localized, but not inside these hyphae. Similarly, the *ha1-1* mutant displayed DAPI-polyP signals in fine branch modules in the central region of the arbuscule and cell walls of the trunk hyphae.

### PolyP Localization in Arbuscules – PPBD Affinity Labeling

**Figure 4:**
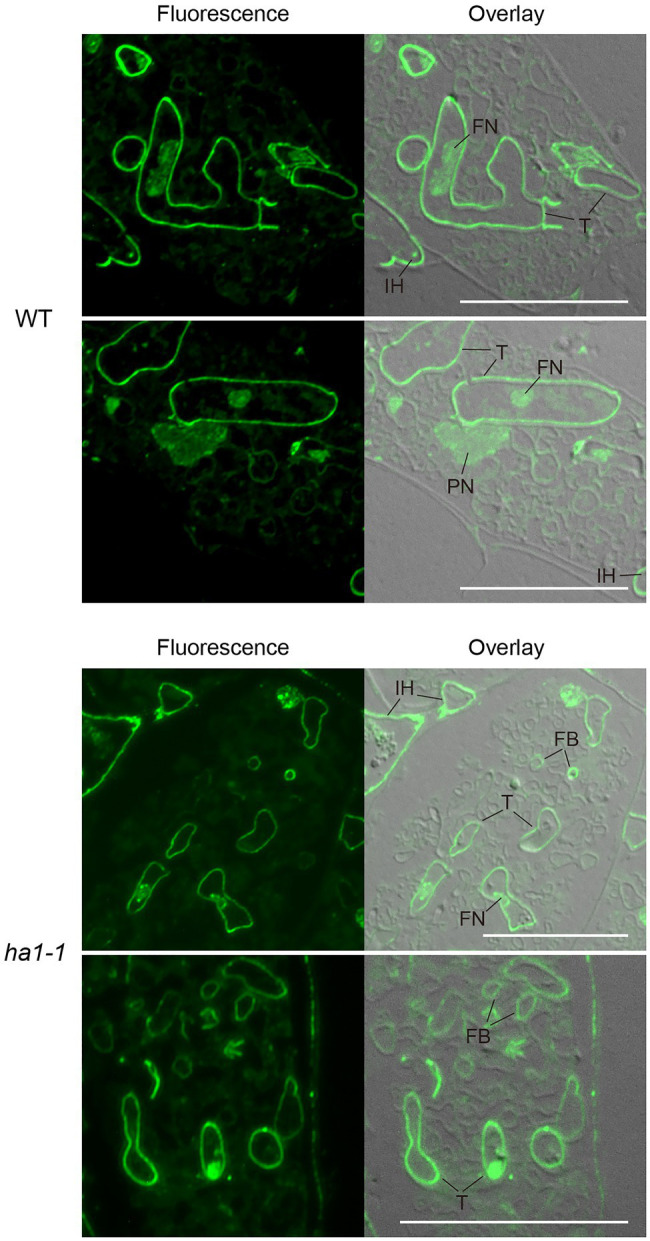
Subcellular localization of polyP using polyphosphate (polyP) binding domain (PPBD) affinity labeling. Representative fluorescence images of polyP localization (left panels) and superimposed differential interference contrast (DIC) images (right panels) of arbuscule-containing cortical cells in wild-type and *ha1-1*. Sections were incubated with a PPBD-anti-Xpress antibody complex and, subsequently, an anti-mouse IgG antibody conjugated with Alexa Fluor 488. An intense fluorescence signal was observed in cell walls of trunk hyphae and intraradical hyphae in both genotypes. Signals were sometimes detected in fine branches in the mutant. FB, fine branch; FN, fungal nucleus; IH, intraradical hypha; PN, plant nucleus; and T, trunk hypha. Scale bars=20μM.

We further analyzed the subcellular localization of polyP using enzyme-affinity labeling with PPBD, which binds specifically to long-chain polyP (Saito et al., 2005), because the yellow fluorescence of DAPI is not specific only to polyP. PolyP was labeled in semithin sections of mycorrhizal roots with recombinant PPBD containing an epitope tag. The tag was then detected by indirect immunocytochemistry with an Alexa Fluor 488-conjugated secondary antibody and observed under a fluorescence microscope. In arbuscules formed in WT roots, a strong polyP signal was observed at the cell periphery of the trunk hyphae, possibly at the cell wall (Figure 4). Weak fluorescence was detected in plant and fungal nuclei, likely due to the low-affinity DNA-binding capability of PPBD (Saito et al., 2005). There was no or very weak polyP signal in the fine branches throughout the cortical cells harboring arbuscules. This localization differed from the DAPI-polyP signal, which was associated with fine branch modules in the central region of arbuscules. These contradictory observations may result from a difference in polyP binding properties as DAPI can bind to short-chain polyP with at least 14 residues (Smith et al., 2018), but PPBD has an extremely low affinity for polyP shorter than 35 residues (Saito et al., 2005). In addition, polyP detected by DAPI was immobilized in ethanol. Conversely, the PPBD enzyme-affinity method included incubation in an aqueous solution that might have eluted some polyP from the sections. In the *ha1-1* mutant, the labeling pattern of fungal polyP was similar to that of the WT, in which the cell periphery of trunk hyphae was labeled (Figure 4). However, we found that polyP signals were occasionally present at the cell periphery of some fine branches.

**Figure 5:**
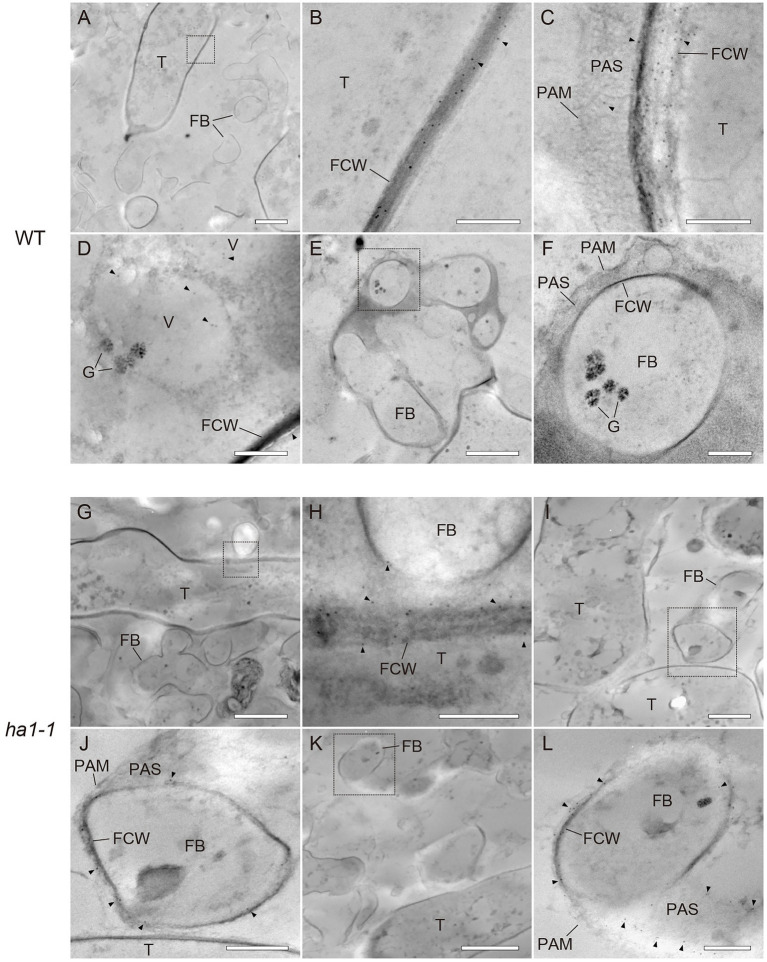
Ultrastructural localization of polyphosphate (polyP) using polyP binding domain (PPBD) affinity labeling. Representative transmission electron micrographs showing polyP distribution (gold particle) in mycorrhizal roots of wild-type **(A–F)** and *ha1-1* **(G–L)**. Sections were incubated with a PPBD-anti-Xpress antibody complex and, subsequently, an anti-mouse IgG conjugated with 6-nm colloidal gold. Triangles show representative gold particles. **(A–C)** PolyP present in the trunk hyphae cell wall. **(D)** Occasional labeling in fungal vacuoles. **(E–F)** Fine branches lacking signal. **(G,H)** Signals were often detected in the trunk hyphae cell walls in the *ha1-1* roots. **(I–L)** Fine branch cell walls were sometimes labeled in *ha1-1*. Panels **(B,F,H,J,L)** show the magnified images of dotted areas in the previous panels. FB, fine branch; FCW, fungal cell wall; G, glycogen granule; PAM, periarbuscular membrane; PAS, periarbuscular space; T, trunk hypha; and V, vacuole. Scale bars=500nm.

PolyP localization was visualized by TEM using the enzyme-affinity method with gold-coupled secondary antibodies. In arbuscules formed in WT, the polyP signal was evenly distributed in the trunk cell wall, as observed with fluorescence microscopy (Figures 5A–C). Sparse labeling was observed sporadically within vacuoles of trunk hyphae (Figure 5D). However, there was no polyP signal in the fine branches (Figures 5E–F). In *ha1-1* roots, polyP localization was similar to that in the WT, with presence in the trunk hyphae fungal cell wall (Figures 5G–H). Interestingly, polyP signals were sometimes observed on the fine branch cell walls (Figures 5I–J). In particular, fine branches cut obliquely against the longitudinal axis showed prominent signals in their cell walls or their surrounding PAS (Figures 5K–L). In the negative controls, where sections of mycorrhizal roots were incubated in a reaction mixture without PPBD, no polyP signals were detected (Supplementary Figure S1). There was also no polyP signal when the PPBD polyP-binding site was masked with a high concentration of tripolyphosphate. In summary, enzyme-affinity labeling using PPBD revealed that in the WT, relatively long-chain polyP was mainly distributed in the trunk hyphae cell walls and absent in the fine branches. However, the *HA1* mutation led to polyP accumulation in some fine branch cell walls.

### Localization of Phosphatase Activity in Arbuscule-Containing Cortical Cells

**Figure 6:**
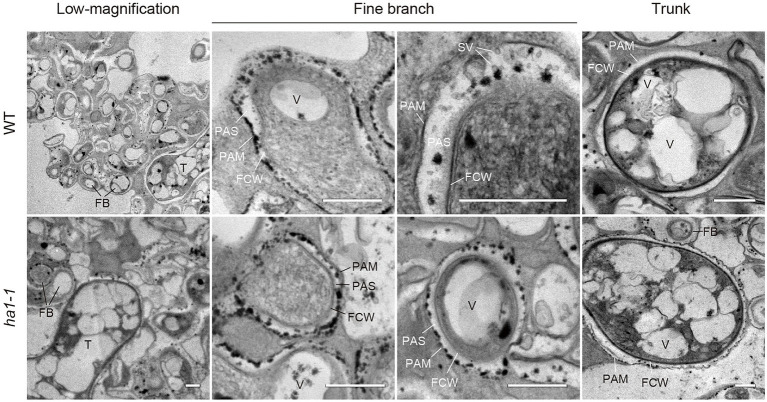
Electron microscopic enzyme cytochemistry of acid phosphatase (ACP) activity in arbuscule-containing cortical cells. Representative transmission electron micrographs showing ACP activity in arbuscule-containing cortical cells of wild-type and *ha1-1* roots colonized by *R. irregularis*. Fine branches and trunk hyphae of an arbuscule colonized in a cortical cell are shown. ACP activity was detected as black precipitates of the reaction product (cerium phosphate) by incubating mycorrhizal roots in acetate buffer (pH 4.6) containing β-glycerophosphate and cerium chloride. FB, fine branch; FCW, fungal cell wall; PAM, periarbuscular membrane; PAS, periarbuscular space; SV, small vesicle; T, trunk hypha; and V, vacuole. Scale bars=500nm.

**Figure 7:**
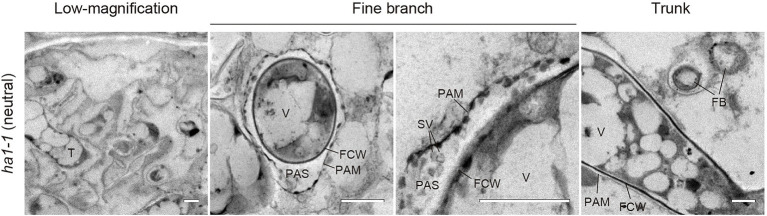
Electron microscopic enzyme cytochemistry of neutral phosphatase (NTP) activity. Transmission electron micrographs showing NTP activity in arbuscule-containing cortical cells of the *ha1-1* roots colonized by *R. irregularis*. NTP activity was detected as black precipitates of the reaction product (cerium phosphate) by incubating mycorrhizal roots in Tris-HCl buffer (pH 7.4) containing β-glycerophosphate and cerium chloride. Fine branches and trunk hyphae of an arbuscule colonized in a cortical cell are shown. FB, fine branch; FCW, fungal cell wall; PAM, periarbuscular membrane; PAS, periarbuscular space; SV, small vesicle; T, trunk hypha; and V, vacuole. Scale bars=500nm.

An interesting feature of polyP localization was its almost complete absence from fine branches at the periphery of cortical cells in WT and *ha1-1* (Figure 3). Dreyer et al. (2008) reported that ACP activity localizes at the interface between fungal cell walls and PAM. Generally, ACPs have broad substrate specificities for phosphate esters. To elucidate the spatial relationship between polyP and ACP in cells with arbuscules, we investigated the localization of ACP activity at the ultrastructural level (Figure 6). ACP activity was detected by incubating mycorrhizal roots in an acidic buffer containing the reaction substrate (β-glycerophosphate) and the co-precipitant (cerium salt) and observing the black precipitates of the reaction product (cerium phosphate) with TEM. High ACP activity was frequently detected in PAS around the fine branches in both WT and *ha1-1*, which was consistent with previous observations (Dreyer et al., 2008). Notably, most of the dense precipitates localized along the host-derived PAM and in small vesicles resembling the intramatrix compartment type I (IMC-I) or apoplastic vesicular structures (AVS; Ivanov et al., 2019; Roth et al., 2019). In contrast, only a few signals were associated with PAS around the trunk hyphae in both WT and *ha1-1* cells. ACP activity was sometimes observed in fungal vacuoles. No dense cerium phosphate precipitate was observed in the control experiment lacking cerium salt (Supplementary Figure S2). In the control reaction without β-glycerophosphate, a few precipitates were observed in the fungal vacuoles, possibly due to a reaction with endogenous vacuole substrates. Next, we investigated the localization of NTP activity in the *ha1-1* mutant because the acidification around arbuscules diminishes in *ha1* cortical cells (Krajinski et al., 2014; Liu et al., 2020). NTP activity localization was similar to ACP activity with signals along the PAM and in small vesicles present in the PAS surrounding fine branches (Figure 7), as previously reported (Jeanmaire et al., 1985).

**Figure 8:**
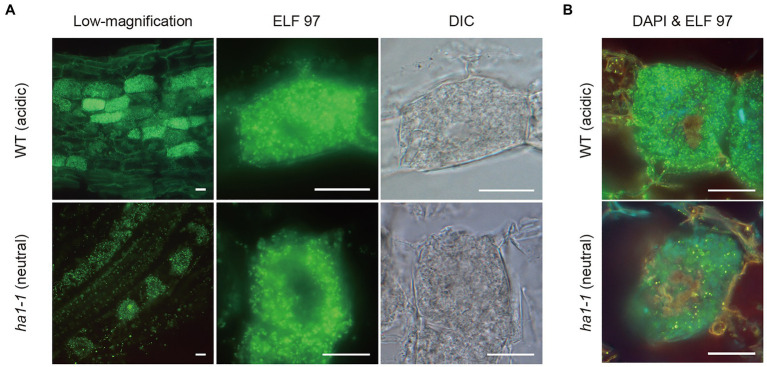
Enzyme cytochemistry of phosphatase activity in arbuscule-containing cortical cells using ELF97. **(A)** Representative fluorescence images of acid and neutral phosphatase activity in wild-type (WT) and *ha1-1*, respectively. Green fluorescence of the ELF97 reaction product indicates phosphatase activity. Arbuscule structure was observed under a DIC microscope. **(B)** Double labeling using DAPI and ELF97 of WT and *ha1-1* under acidic and neutral conditions, respectively. The color balance was adjusted to discriminate between DAPI-polyP complex and the ELF97 reaction product fluorescence. Yellow-orange and green fluorescence indicate polyP and phosphatase activity, respectively. Scale bars=20μM.

To further study the relationship between phosphatase activity and polyP accumulation, we detected phosphatase activity and polyP signals simultaneously by enzyme cytochemistry using the ELF97 phosphatase substrate and DAPI staining, respectively. ELF97 forms fine precipitates after hydrolysis of its phosphate ester bond by non-specific phosphatases, emitting yellow-green fluorescence at the site of phosphatase activity (Haugland, 2005). First, ACP and NTP activities were visualized in a typical mature arbuscule by single ELF97 staining in WT and *ha1-1* roots, respectively (Figure 8A). In the WT, ACP activity was present throughout the arbuscule but was excluded from its central region. Similarly, NTP activity was detected in arbuscules in the mutant but the central region without phosphatase activity was larger than that in the WT. Next, we performed double labeling of phosphatase activity and polyP. The localization of phosphatase activity (green) and polyP (yellow) was distinct based on different emission colors using a long-pass filter, albeit showing weak and different color signals compared to the single staining (Figure 8B). Double labeling showed that polyP was present in the center of arbuscules, and phosphatase activity localized in the surrounding regions. Thus, polyP and phosphatase activity showed opposite localization in both WT and *ha1-1* cells.

### Gene Expression Analysis

**Figure 9:**
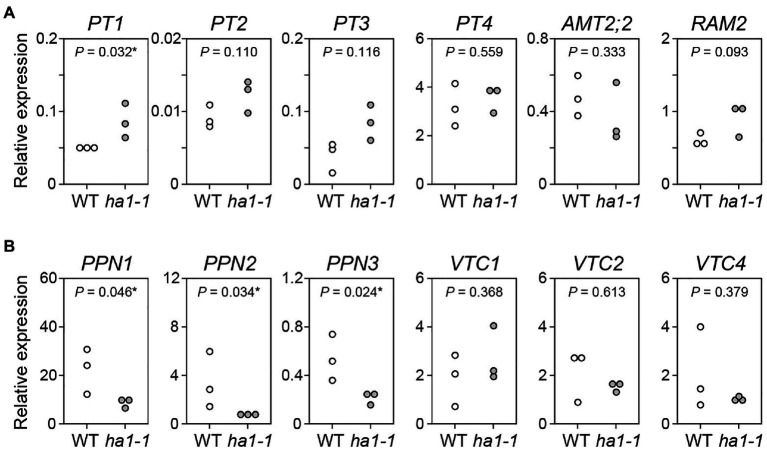
Gene expression analysis. **(A)** Expression of *L. japonicus* genes encoding phosphate transporters (*PT1*–*PT4*), ammonium transporter (*AMT2;2*), and AM-specific glycerol-3-phosphate acyltransferase *RAM2* gene in mycorrhizal roots of the wild-type and *ha1-1* 4weeks after inoculation with *R. irregularis*. Expression levels were normalized based on the amount of *L. japonicus EF2*. **(B)** Expression of *R. irregularis* genes encoding endopolyphosphatases (*PPN1*–*PPN3*) and vacuolar transporter chaperones (*VTC1*, *VTC2*, and *VTC4*) in mycorrhizal roots. The *R. irregularis EF1β* gene was used as an internal control. *p*-values are based on Student’s *t*-test (^*^, *p*<0.05).

We investigated whether the *HA1* mutation affected gene expression related to plant Pi uptake and fungal polyP metabolism during AM symbiosis. The levels of the phosphate transporter *PT1*, likely to function in the direct pathway due to its downregulation during mycorrhization (Maeda et al., 2006), were slightly elevated in *ha1-1* roots compared to the WT (Figure 9A), which may reflect a partial block in P translocation *via* the mycorrhizal pathway. Transcripts of other phosphate transporters, including AM-specific *PT4* (Guether et al., 2009a; Takeda et al., 2009), accumulated equally in the mycorrhizal roots of both genotypes. Similarly, the *ha1-1* mutation did not affect the expression of AM marker genes, AM-specific ammonium transporter *AMT2;2* (Guether et al., 2009b) and glycerol-3-phosphate acyltransferase *RAM2* (Wang et al., 2012). Fungal endopolyphosphatase genes *PPN1*, *PPN2*, and *PPN3* were downregulated in AM fungi colonizing the mutant, whereas the VTC genes involved in polyP synthesis were not affected (Figure 9B).

## Discussion

P transfer across the symbiotic interface is an important process in the mycorrhizal pathway. However, it has been challenging to elucidate this process, possibly due to rapid P movement from the AM fungus to the host. In this study, we sought to clarify the intermediate process of P transfer by visualizing polyP localization in arbuscules of *R. irregularis* formed in *L. japonicus* WT and the *ha1-1* mutant.

PPBD affinity-labeling for specifically detecting long-chain polyP demonstrated that polyP was predominantly distributed in cell walls of trunk hyphae in arbuscules formed in WT, but fine branch cell walls lacked a polyP signal. Since intense ACP activities were found in PAS around fine branches, the absence of polyP in the fine branch cell walls could be explained by the degradation of polyP in fungal cell walls by apoplastic ACP. Supporting this idea, the *ha1-1* mutant, in which the mycorrhizal pathway was partially suppressed, showed polyP localization in some cell walls of the fine branches. Based on our observations, we propose a hypothesis for P transfer at AM fungus-host interface in which polyP is released into the cell walls of fine branches and then immediately subjected to hydrolysis by ACP located in the PAS. The liberated Pi is delivered to host cells by symbiotic Pi transporters driven by the H^+^ gradient generated across the PAM by the HA1 H^+^-ATPase (Javot et al., 2007; Yang et al., 2012; Willmann et al., 2013; Xie et al., 2013; Krajinski et al., 2014; Wang et al., 2014; Watts-Williams et al., 2015; Liu et al., 2020). However, because the mechanism of polyP release into the fungal cell wall is unknown and it remains unclear whether the apoplastic ACP can catalyze polyP hydrolysis, we cannot rule out the possibility that polyP is hydrolyzed in AM fungal hyphae and the liberated Pi is exported to the PAS *via* an unidentified Pi exporter.

Moreover, polyP signals were observed within several fine branch modules close to the trunk hyphae by DAPI staining. The fine branch modules with polyP were slightly expanded in the *ha1-1* mutant. Around these fine branch modules, no phosphatase activity was observed by double staining with ELF97 and DAPI. It might be anticipated that P metabolism and export are suppressed in these fine branch modules, leading to the accumulation of polyP. This is consistent with our finding that the expression levels of endopolyphosphatase genes were reduced in AM fungi colonizing the *ha1-1* mutant, although whether polyphosphatase activity is decreased in DAPI-stained fine branch modules stained is unknown.

A remarkable feature of arbuscule polyP is its localization in the fungal cell walls. The polyP chain appears to be relatively long, as detected by the PPBD enzyme affinity method. PolyP distribution in cell walls has also been observed in germ tubes and extraradical hyphae of AM fungi and mycelia of a wide range of fungal species (Mucoromycota, Dikarya, and Chytridiomyceta; Werner et al., 2007; Kuga et al., 2008). However, little is known about the molecular mechanisms underlying polyP accumulation in fungal cell walls. A possible mechanism is that polyP might be loaded into intracellular vesicles and released into the extracellular space *via* exocytosis. Alternatively, the VTC complex might be redistributed to the plasma membrane and then synthesize polyP. The yeast VTC2, a subunit of VTC complexes, is observed at the cell periphery along the plasma membrane under high Pi conditions, although it is localized in vacuoles in a low-Pi medium (Hothorn et al., 2009). How polyP is released across the fungal plasma membrane and the role of cell wall polyP in fungal physiology are important questions to be explored in future studies.

Extensive polyP accumulation in arbuscules has been observed in plant mutants of Pi transporter genes related to symbiotic P uptake. Arbuscules formed in *M. truncatula PT4* mutant and RNAi lines (Javot et al., 2007) and in *L. japonicus PT3* RNAi lines (Funamoto et al., 2007) were almost entirely stained with toluidine blue and DAPI, respectively, indicating that polyP accumulation extended to fine branches in the marginal regions of the arbuscule. However, the *ha1-1* mutant had DAPI-polyP signals in only some fine branch modules located at the arbuscule center. Similarly, *ha1-1* phenotypes in P uptake through the mycorrhizal pathway and early arbuscule degradation were not as severe as in *pt* or other *ha1* mutants (Javot et al., 2007; Krajinski et al., 2014; Wang et al., 2014; Liu et al., 2020). The *ha1-1* mutation may have partial ATPase activity. In both *ha1-1* and WT roots, arbuscule fine branch modules close to a trunk hypha displayed strong DAPI-polyP fluorescent signals but were not labeled with PPBD. Considering the differences in polyP chain length affinity between DAPI and PPBD (Saito et al., 2005; Smith et al., 2018), relatively short-chain polyPs are likely to accumulate in that region. In the *ha1-1* mutant, fine branch modules stained with DAPI were increased and expanded to the periphery of arbuscules. A similar pattern of polyP accumulation was observed in RNAi lines of the AM-inducible Pi transporter *PT3* (Funamoto et al., 2007). The P flow in arbuscules appear to be initially disrupted in the fine branches close to the trunk hypha, which is observed as polyP accumulation. Subsequently, the disruption of the P flow may be extended to fine branches in other regions.

We also found that ACP activity and polyP have opposite localization in mature arbuscules. Funamoto et al. (2007) observed a similar spatial relationship between alkaline phosphatase (ALP) activity and polyP distribution. ALPs in AM fungi (Gianinazzi-Pearson and Gianinazzi, 1978; Kojima et al., 1998; Aono et al., 2004) can hydrolyze monophosphate esters but not pyrophosphate bonds in polyP (Ezawa et al., 1999; Liu et al., 2013). Therefore, the reduced accumulation of polyP at sites of intense ALP activity could result from a change in P metabolism indirectly mediated by fungal ALPs (Saito and Ezawa, 2016). ACP has a broad substrate specificity for various phosphate compounds. Some plant ACPs can potentially hydrolyze polyP (Ezawa and Yoshida, 1994; Ezawa et al., 2005; Huang et al., 2018). Enzyme histochemical analyses have demonstrated intense ACP activity in arbuscules, particularly in PAM (Van Aarle et al., 2005; Dreyer et al., 2008). In our study, ACP activity detected in PAS was often associated with host-derived PAM and small vesicles resembling IMC-I or AVS, possibly due to PAM outgrowth (Ivanov et al., 2019; Roth et al., 2019). This observation suggests that ACP in the apoplastic region originates from the plant cells. However, the corresponding proteins have not been identified in the host plants. Several AM-inducible phosphatase genes are candidates for encoding ACPs present in PAS. In soybean, two out of 35 purple ACP genes are upregulated in AM roots (Li et al., 2012). The AM-inducible soybean purple ACP gene, *GmPAP33*, is expressed in arbuscule-containing cortical cells and is involved in arbuscule degeneration *via* phospholipid hydrolysis (Li et al., 2019). The AM-inducible marigold purple ACP TpPAP1, which differs from GmPAP33 in subclass, displays a broad substrate specificity and can catalyze polyP degradation (Ezawa and Yoshida, 1994; Ezawa et al., 2005). Further research, including mutant analysis, is needed to clarify whether these purple ACPs are responsible for ACP activity in PAS.

## Data Availability Statement

The original contributions presented in the study are included in the article/Supplementary Material, further inquiries can be directed to the corresponding author.

## Author Contributions

CN conducted all experiments and wrote the first draft. CN and KS analyzed the data, designed the experiments, and contributed to the final manuscript. All authors contributed to the article and approved the submitted version.

## Funding

This work was supported by the Science and Technology Research Promotion Program for Agriculture, Forestry, and Fisheries, the Food industry (26036A) from the Ministry of Agriculture, Forestry, and Fisheries of Japan (KS), and a Grant-in-Aid for Scientific Research (15H01751) from the Japan Society for the Promotion of Science (KS).

## Conflict of Interest

The authors declare that the research was conducted in the absence of any commercial or financial relationships that could be construed as a potential conflict of interest.

## Publisher’s Note

All claims expressed in this article are solely those of the authors and do not necessarily represent those of their affiliated organizations, or those of the publisher, the editors and the reviewers. Any product that may be evaluated in this article, or claim that may be made by its manufacturer, is not guaranteed or endorsed by the publisher.

## Footnotes

^1^ https://lotus.au.dk/
