# Supplementary material for: Role of Cell Wall Polyphosphates in Phosphorus Transfer at the Arbuscular Interface in Mycorrhizas
Source: Front Plant Sci. 2021 Sep 20;12:725939. doi: 10.3389/fpls.2021.725939 (PMC8488203; doi:10.3389/fpls.2021.725939)
Supplement: Supplementary file 1 [file Data_Sheet_1.PDF]

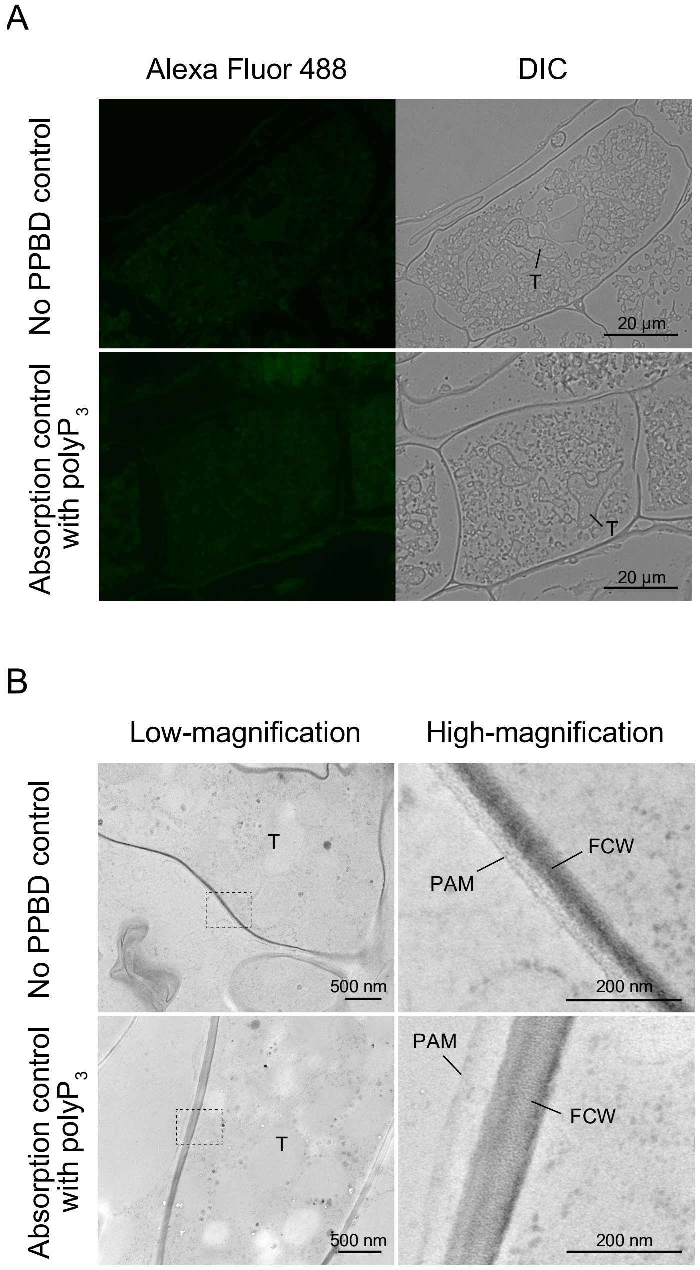

Supplementary Figure 1 | Negative controls for polyphosphate (polyP) labeling using the polyP binding domain (PPBD) affinity method. Sections of wild-type (WT) *Lotus japonicus* roots colonized by *Rhizophagus irregularis* were incubated without PPBD (no PPBD control) or with an excess of tripolyphosphate (absorption control with polyP$_3$) during the PPBD-anti-Xpress antibody incubation. **(A)** Fluorescence microscopy images of Alexa Fluor 488, a secondary antibody conjugate, and differential interference contrast (DIC) images of arbuscules. **(B)** Micrographs of transmission electron microscopy. The right panels show magnified images of the dotted areas in the left panels. FCW: fungal cell wall, PAM: periarbuscular membrane, T: trunk hypha.

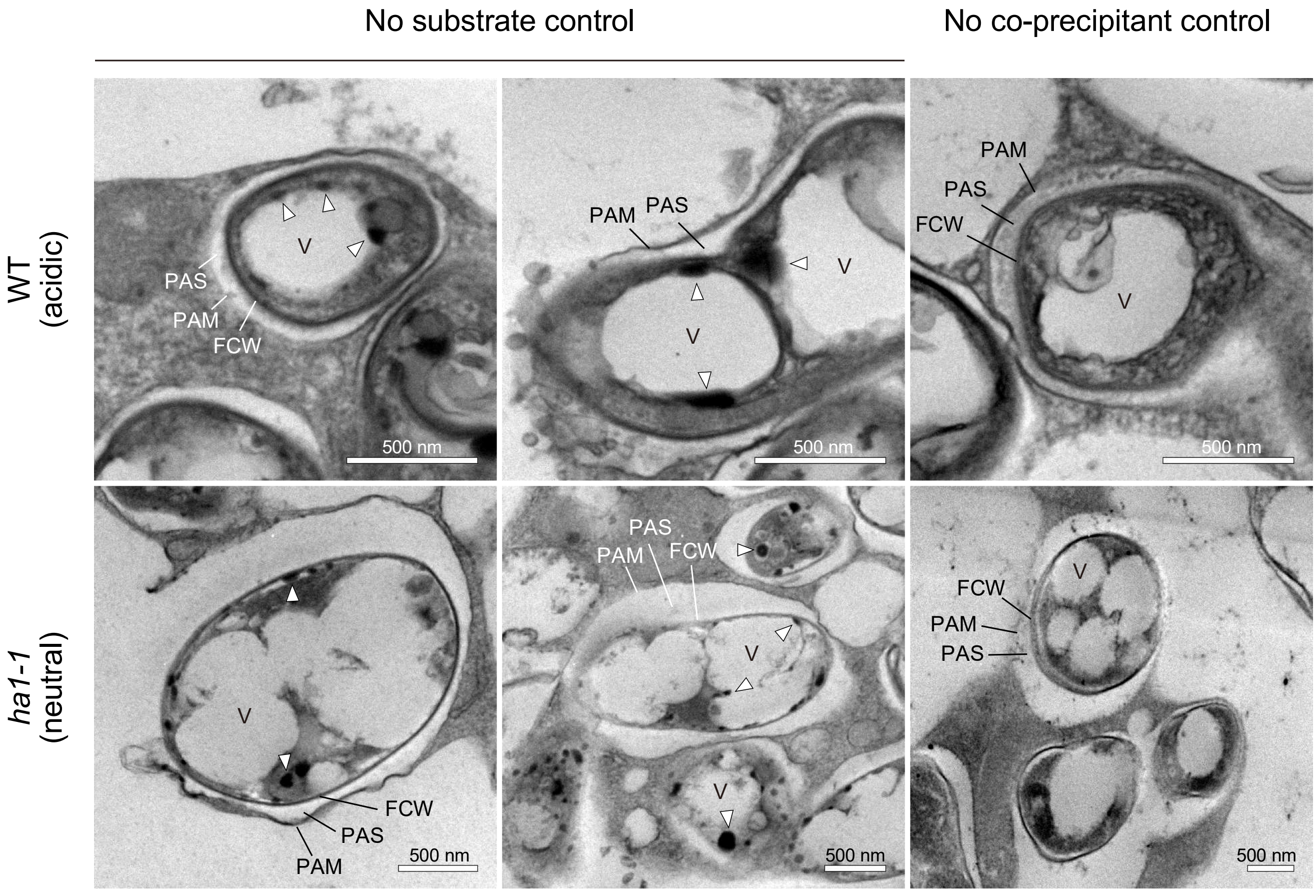

Supplementary Figure 2 | Negative controls for enzyme cytochemistry of phosphatase activity. Transmission electron micrographs show acid and neutral phosphatase activity in arbuscule-containing cortical cells of wild-type (WT) and *ha1-1*, respectively. Mycorrhizal roots were incubated in the absence of β-glycerophosphate (no substrate control) or cerium salt (no co-precipitant control) in the reaction buffer. In the no substrate control, a few precipitates (white triangles) were observed in fungal vacuoles. FCW: fungal cell wall, PAM: periarbuscular membrane, PAS: periarbuscular space, V: vacuole.

Supplementary Table 1 | Primers used in this study.

Gene name	Primer sequence (5'→3')	Reference
**Screening of *LORE1* insertion mutants**		
HA1 (plant ID: 30006854)	TTTCATTGTGGAACTGGACATTTTGA	This study
	TGGTTCACATAGGCTGTCTCAGCA	
P2 primer	ACCTTGTTGCTTCAGCCATGG	Urbanski *et al.* 2012.
**RT-PCR**		
HA1	AAGCGCGGAGATTATCAAGA	This study
	TTTACCACCAGATCCCAAGC	
EF2	AGTTGCTTTGGTTGGTTTGG	This study
	AAGTGGGGTACCTTGCCTCT	
**qRT-PCR**		
Lotus japonicus		
EF2	GGTGGCTGCTGCTGGAATTA	This study
	ACGGGTATCAGTCATACGGACAT	
PT1	TGCCGGCCGAGATTTTC	This study
	GCAGCTGAGATTCCATGACAAG	
PT2	GGGCCTAATGCCACCACAT	This study
	CGGAATCTAGCCGGGAAAA	
PT3	CCTCACGCGGAGGAAATG	This study
	GTTGATGCTCTTGCAACCTTGTA	
PT4	TCCGGGCTCTCCTTTGG	Kojima *et al.* 2014.
	AGAAGCATAGCGTTCCCATCA	
AMT2;2	ACACATGCTTGCACTGCTACC	Guether *et al.* 2009.
	CTGCCCATCCTTGAACAACCC	
RAM2	GCCCGTAGCCATGGTGAA	This study
	CCCTCTTGCAGTGGTTCCA	
Rhizophagus irregularis		
EF1β	CCCATGCAGCTCGATGGTA	Kobae *et al.* 2015.
	TGCCAGGAAGTGAAGAAAATGA	
VTC1	GAGAATCAGGTCCTTATGATGATAGGT	This study
	CACCCACGAGGAAAAAGCA	
VTC2	GCACGACAGACGTACAAGTTACAA	This study
	AATGGGCACGCGTTCAAA	
VTC4	CATGGTGTCGCAACACTTATGG	This study
	GTGGAAGCCAGAAAGGAAAAAG	
PPN1	GAAAGATTTAACAATACCGAGTTGGAT	This study
	TGCGCTATTAAGTCCATCATTAACA	
PPN2	TCGACCACCTAATGCTTTTGG	This study
	ATCGCAATTATATTCTCCCCATTTT	
PPN3	CCGAATAGTGACCCATCCAAA	This study
	CATTCCGATTGTGGAGTTCCA	

Guether et al. 2009. Plant Physiology 150: 73–83; Kobae et al. 2015. Mycorrhiza 25: 411–417;
Kojima et al. 2014. Plant & Cell Physiology 55: 928–941; Urbański et al. 2012. Plant Journal 69: 731–741.
